# Supplementary material for: The sex of organ geometry
Source: Nature. 2024 May 29;630(8016):392–400. doi: 10.1038/s41586-024-07463-4 (PMC11168936; doi:10.1038/s41586-024-07463-4)
Supplement: Supplementary file 1 — List of full genotypes, sample sizes, P values and statistical tests used and tables of organ contact frequencies. [file 41586_2024_7463_MOESM1_ESM.docx]

**Supplementary information**

**Supplementary information guide**

1. List of full genotypes

2. Sample sizes, p-values and statistical tests used

3. Organ contact frequencies

**1. List of full genotypes**

**Figure 1**

a-b, d-r)

*OregonR*

**Figure 2**

a)

*w^[*]^/w^[1118]^; DSRF-gal4, 5xUAS-CD8::GFP/ +; 10xUAS-CD8::GFP/ +*

b)

*OregonR*

*w^[*]^/w^[1118]^; btl-Gal4, UAS-myr(src42A)::GFP/ +*

c)

*w^[*]^/w^[1118]^;; trh(GMR14D03)-Gal4 / 10xUAS-IVS-myr::GFP*

d)

*w^[*]^/w^[1118]^; 10xUAS-CD8::GFP/ + ; trh(GMR14D03)-Gal4/ +*

*w^[*]^/w^[1118]^; UAS-Bax, tub-Gal80^TS^/ 10xUAS-CD8::GFP; trh(GMR14D03)-Gal4/ +*

e-g)

*w^[*]^/w^[1118]^;; trh(GMR14D03)-Gal4/ +*

*w^[*]^/w^[1118]^; UAS-Bax, tub-Gal80^TS^/ +; trh(GMR14D03)-Gal4/+*

h)

*w^[*]^/w^[1118]^; Hand-Gal4^[MI04106-TG4.0]^/ +; QF6, QUASmtdTomato/ +*

*w^[*]^/w^[1118]^; Hand-Gal4^[MI04106-TG4.0]^/ + ; QF6, QUASmtdTomato / UAS-bnlRNAi.GD3070*

i-k)

*w^[*]^/w^[1118]^; Hand-Gal4^[MI04106-TG4.0]^/ +;*

*w^[*]^/w^[1118]^; Hand-Gal4^[MI04106-TG4.0]^/ + ; UAS-bnlRNAi.GD3070/ +*

**Figure 3**

a)

*w^[*]^/w^[1118]^;; bnl^[lexA]^, 13XLexAop2-IVS-myr::GFP/ +*

b)

*w^[*]^, Su(H)GBE-LacZ/ w^[1118]^; esg-Gal4 NP7397, UAS-GFP, Tub-Gal80^TS^/ +*

c)

*OregonR*

d)

*w^[*]^/w^[1118]^; btl-Gal4, UAS-myr(src42A)::GFP/ +*

**Figure 4**

a-f)

*w^[*]^; Hand-Gal4^[MI04106-TG4.0]^/ +; bnl^[lexA]^, 13XLexAop2-IVS-myr::GFP/ UAS-mCherry.RNAi.Valium10*

*w^[*]^; Hand-Gal4^[MI04106-TG4.0]^/ +; bnl^[lexA]^, 13XLexAop2-IVS-myr::GFP/ UAS-SxlRNAi.TRiPGL00634*

*w^[*]^; Hand-Gal4^[MI04106-TG4.0]^/ UAS-mCherry.RNAi.Valium10;*

*w^[*]^; Hand-Gal4^[MI04106-TG4.0]^/ UAS-SxlRNAi.TRiPGL00634;*

g-l)

*w^[*]^; Hand-Gal4^[MI04106-TG4.0]^/ +; bnl^[lexA]^, 13XLexAop2-IVS-myr::GFP/ +*

*w^[*]^; Hand-Gal4^[MI04106-TG4.0]^/ +; bnl^[lexA]^, 13XLexAop2-IVS-myr::GFP/ UAS-Sxl.alt5-C8*

*w^[*]^; Hand-Gal4^[MI04106-TG4.0]^/ +; UAS-mCherry.RNAi.Valium10/ +*

*w^[*]^; Hand-Gal4^[MI04106-TG4.0]^/ +; UAS-Sxl.alt5-C8/ +*

**Figure 5**

a-b)

*w^[*]^/w^[1118]^; Hand-Gal4^[MI04106-TG4.0]^/ +; Ubi-EGFP.ODD, Ubi-mRFP.nls/ +*

*w^[*]^/w^[1118]^; Hand-Gal4^[MI04106-TG4.0]^/ +; UAS-bnlRNAi.GD3070/ Ubi-EGFP.ODD, Ubi-mRFP.nls*

*w^[*]^/w^[1118]^; Hand-Gal4^[MI04106-TG4.0]^/ +; Ldh::GFP^[YD0852]^/ +*

*w^[*]^/w^[1118]^; Hand-Gal4^[MI04106-TG4.0]^/ +; UAS-bnlRNAi.GD3070/ Ldh::GFP^[YD0852]^*

c-e)

*w^[*]^/w^[1118]^; Hand-Gal4^[MI04106-TG4.0]^/ +;*

*w^[*]^/w^[1118]^;; UAS-bnlRNAi.GD3070/ +*

*w^[*]^/w^[1118]^; Hand-Gal4^[MI04106-TG4.0]^/ +; UAS-bnlRNAi.GD3070/ +*

f)

*w^[*]^/w^[1118]^; Hand-Gal4^[MI04106-TG4.0]^/ UAS-mCherry.RNAi.Valium10;*

*w^[*]^/w^[1118]^; UAS-SxlRNAi.TRiPGL00634/ +;*

*w^[*]^/w^[1118]^; Hand-Gal4^[MI04106-TG4.0]^/ UAS-SxlRNAi.TRiPGL00634;*

**Extended Data Figure 1**

a-b) , i-k)

*Canton-S*

c-h), s-v)

*OregonR*

l-n)

*w^[1118]^; Hand-Gal4^[MI04106-TG4.0]^/ +;*

o-r)

*OregonR*

*ovo^D1^/ +*

**Extended Data Figure 2**

a-i), m-r)

*OregonR*

j-l)

*OregonR*

*ovo^D1^/ +*

**Extended Data Figure 3**

a-c)

*OregonR*

*w^[*]^/w^[1118]^; btl-Gal4, UAS-myr(src42A)::GFP/ +*

d)

*w^[*]^/w^[1118]^; 10xUAS-CD8::GFP/ + ; trh(GMR14D03)-Gal4/ +*

*w^[*]^/w^[1118]^; UAS-Bax, tub-Gal80^TS^/ 10xUAS-CD8::GFP; trh(GMR14D03)-Gal4/ +*

*w^[*]^/w^[1118]^;; trh(GMR14D03)-Gal4/ +*

*w^[*]^/w^[1118]^; UAS-Bax, tub-Gal80^TS^/ +; trh(GMR14D03)-Gal4/ +*

e)

*w^[*]^/w^[1118]^; Hand-Gal4^[MI04106-TG4.0]^/ +; QF6, QUAS-mtdTomato/ +*

*w^[*]^/w^[1118]^; Hand-Gal4^[MI04106-TG4.0]^/ +; QF6, QUAS-mtdTomato/ UAS-bnlRNAi.GD3070*

*w^[*]^/w^[1118]^; Hand-Gal4^[MI04106-TG4.0]^/ +;*

*w^[*]^/w^[1118]^; Hand-Gal4^[MI04106-TG4.0]^/ + ; UAS-bnlRNAi.GD3070/ +*

f)

*w^[*]^/w^[1118]^; Hand-Gal4^[MI04106-TG4.0]^/ tub-Gal80.ts; QF6, QUAS-mtdTomato/ +*

*w^[*]^/w^[1118]^; Hand-Gal4^[MI04106-TG4.0]^/ tub-Gal80.ts; QF6, QUAS-mtdTomato/ UAS-bnlRNAi.GD3070*

g-k)

*w^[*]^/w^[1118]^; vm(GMR13B09)-Gal4/ +;*

*w^[*]^/w^[1118]^; vm(GMR13B09)-Gal4/ +; UAS-bnlRNAi.GD3070/ +*

**Extended Data Figure 4**

a), c-d)

*w^[*]^/w^[1118]^;; bnl-Gal4^[MI00874- TG4.1]^, UAS-StingerGFP / +*

b)

*w^[*]^/w^[1118]^;; bnl^[lexA]^, 13XLexAop2-IVS-myr::GFP/ +*

*OregonR*

e)

*w^[*]^/w^[1118^;Hand-Gal4^[MI04106-TG4.0]^/ UAS-myr(src42A)::GFP;*

f)

*w^[*]^/w^[1118]^; UAS-myr(src42A)::GFP/ +; vm(GMR13B09)-Gal4/ +*

**Extended Data Figure 5**

a-b) , e-f)

*w^[*]^/w^[1118]^; mex1-Gal4 /+; QF6, QUAS-mtdTomato*/ *+*

*w^[*]^/w^[1118]^; mex1-Gal4/ +; QF6, QUAS-mtdTomato*/ *UAS-bnlRNAi.GD3070*

*w^[*]^/w^[1118]^; mex1-Gal4 /+;*

*w^[*]^/w^[1118]^; mex1-Gal4/ +; UAS-bnlRNAi.GD3070/ +*

c-d), g-h)

*w^[*]^/w^[1118]^; mex1-Gal4, esg^[NP7397]^-Gal4/ tub-Gal80.ts; QF6, QUAS-mtdTomato*/ *+*

*w^[*]^/w^[1118]^; mex1-Gal4, esg^[NP7397]^-Gal4/ tub-Gal80.ts; QF6, QUAS-mtdTomato*/ *UAS-btlRNAi.KK100331*

*w^[*]^/w^[1118]^; mex1-Gal4, esg^[NP7397]^-Gal4/ tub-Gal80.ts;*

*w^[*]^/w^[1118]^; mex1-Gal4, esg^[NP7397]^-Gal4/ tub-Gal80.ts; UAS-btlRNAi.KK100331/ +*

**Extended Data Figure 6**

a)

*w^[1118]^*

*w^[*]^/w^[1118]^; btl-Gal4/ +; UAS-StingerGFP/ +*

b)

*w^[*/^w^[1118]^;; QF6, QUAS-mtdTomato/ +*

*w^[*]^/w^[1118]^; DSRF-gal4, 5xUAS-CD8::GFP/ +; 10xUAS-CD8::GFP/ +*

*w^[*]^; 10xUAS-CD8::GFP/ +; trh(GMR14D03)-Gal4/ +*

**Extended Data Figure 7**

a-b)

*y^[1]^, w^[*]^/y^[1]^, v^[1]^; btl-Gal4, UAS-myrGFP/ +; UAS-mCherry.RNAi.Valium10/ +*

*y^[1]^, w^[*]^/y^[1]^, v^[1]^; btl-Gal4, UAS-myrGFP/ +; UAS-traRNAi.TRiPJF03132/ +*

*y^[1]^, w^[*]^/y^[1]^, v^[1]^; btl-Gal4/ +; UAS-mCherry.RNAi.Valium10/ +*

*y^[1]^, w^[*]^/y^[1]^, v^[1]^; btl-Gal4/ +; UAS-traRNAi.TRiPJF03132/ +*

c-g)

*w^[*]^/w^[1118]^; btl-Gal4, UAS-myr(src42A)::GFP/ +;*

*w^[*]^/w^[1118]^; btl-Gal4, UAS-myrGFP; UAS-traRNAi.GD764*

*y^[1]^, w^[*]^/w^[1118]^; btl-Gal4/+*

*y^[1]^, w^[*]^/w^[1118]^; btl-Gal4/ +; UAS-traRNAi.GD764/ +*

**Extended Data Figure 8**

a-g)

*w^[*]^; Hand-Gal4^[MI04106-TG4.0]^/ UAS-Dcr-2; bnl^[lexA]^, 13XLexAop2-IVS-myr::GFP/ UAS-mCherry.RNAi.Valium10*

*w^[*]^, UAS-Dcr-2; Hand-Gal4^[MI04106-TG4.0]^/ +; bnl^[lexA]^, 13XLexAop2-IVS-myr::GFP/ UAS-traRNAi.TRiPJF03132*

*w^[*]^; Hand-Gal4^[MI04106-TG4.0]^/ UAS-Dcr-2; QF6, QUAS-mtdTomato*/ *UAS-mCherry.RNAi.Valium10*

*w^[*]^, UAS-Dcr-2; Hand-Gal4^[MI04106-TG4.0]^/ +; QF6, QUAS-mtdTomato*/ *UAS-traRNAi.TRiPJF03132*

*w^[*]^; Hand-Gal4^[MI04106-TG4.0]^/ UAS-Dcr-2; UAS-mCherry.RNAi.Valium10/ +*

*w^[*]^, UAS-Dcr-2; Hand-Gal4^[MI04106-TG4.0]^/ + ; UAS-traRNAi.TRiPJF03132/ +*

h-i)

*w^[*]^; Hand-Gal4^[MI04106-TG4.0]^/ UAS-mCherry.RNAi.Valium10;*

*w^[*]^; Hand-Gal4^[MI04106-TG4.0]^/ UAS-SxlRNAi.TRiPGL00634;*

j-k)

*w^[*]^/w^[1118]^; Hand-Gal4^[MI04106-TG4.0]^/ +;*

*w^[*]^/w^[1118]^; Hand-Gal4^[MI04106-TG4.0]^/ +; UAS-Sxl.alt5-C8/ +*

**Extended Data Figure 9**

a)

*w^[*]^/w^[1118]^;; Ubi-EGFP.ODD, Ubi-mRFP.nls/ +*

b)

*w^[*]^/w^[1118]^; Hand-Gal4^[MI04106-TG4.0]^/ +; Ubi-EGFP.ODD, Ubi-mRFP.nls/ +*

*w^[*]^/w^[1118]^; Hand-Gal4^[MI04106-TG4.0]^/ +; UAS-bnlRNAi.GD3070/ Ubi-EGFP.ODD, Ubi-mRFP.nls*

*w^[*]^/w^[1118]^; Hand-Gal4^[MI04106-TG4.0]^/ +; Ldh::GFP^[YD0852]^/ +*

*w^[*]^/w^[1118]^; Hand-Gal4^[MI04106-TG4.0]^/ +; UAS-bnlRNAi.GD3070/ Ldh::GFP^[YD0852]^*

**Extended Data Figure 10**

a,b), e), g-h)

*w^[*]^/w^[1118]^; Hand-Gal4^[MI04106-TG4.0]^/ +;*

*w^[*]^/w^[1118]^;; UAS-bnlRNAi.GD3070/ +*

*w^[*]^/w^[1118]^; Hand-Gal4^[MI04106-TG4.0]^/ +; UAS-bnlRNAi.GD3070/ +*

c-d), f)

*w^[*]^/w^[1118]^; Hand-Gal4^[MI04106-TG4.0]^/ +;*

*w^[*]^/w^[1118]^; Hand-Gal4^[MI04106-TG4.0]^/ +; UAS-bnlRNAi.GD3070/ +*

**2. Sample sizes, p-values and statistical tests used**

| **Figure** | **Genotype/sex** | **Number of animals used (n)** | **Exact p value and statistical test** |
| --- | --- | --- | --- |
| 1d | *OregonR* Male  *OregonR* Female | 62  60 |  |
| 1e | *OregonR* Male  *OregonR* Female | 62  60 | (Procrustes ANOVA results in Supplementary Tables 1-2) |
| 1f | *OregonR* Male  *OregonR* Female | 62  60 | (Procrustes ANOVA results in Supplementary Tables 3-4) |
| 1g | *OregonR* Male  *OregonR* Female | 44  40 |  |
| 1h | *OregonR* Male  *OregonR* Female | 41  39 | Tilt angle  Two-sample t-test: p=2*10^-5 |
| 1i-j | *OregonR* Male  *OregonR* Female | 41  39 | MDS stats – see Methods  Males vs Females p=1.8*10^-7 |
| 1p | *OregonR* Female | 60 | Ovary Volume  Paired t-test p=1.9*10^-4 |
| 1q | *OregonR* Female | 44 |  |
| 1r | *OregonR* Male | 44 |  |
| 2b | *btl > myrGFP* Female  *OregonR* Female | 21  40 |  |
| 2e | *trh > Ctrl* Male  *trh > Bax* Male  *trh > Ctrl* Female  *trh > Bax* Female | 29  35  14  41 | (Procrustes ANOVA results in Supplementary Tables 20-21) |
| 2f-g | *trh > Ctrl* Male  *trh > Bax* Male  *trh > Ctrl* Female  *trh > Bax* Female | 29  35  14  41 | MDS stats – see Methods  Ctrl vs *Bax* Males p=0.003  Ctrl vs *Bax* Females p=0.002 |
| 2h | *QF6>mtdTomato*  *Hand > Ctrl* Male  *Hand > bnlRNAi* Male  *Hand > Ctrl* Female  *Hand > bnlRNAi* Female  DSRF staining  *Hand > Ctrl* Male  *Hand > bnlRNAi* Male  *Hand > Ctrl* Female  *Hand > bnlRNAi* Female | 12  10  11  10  23  30  27  27 | Total tracheal length:  ANOVA: Total Trachea Length~sex*genotype  Sex p=0.00284  Genotype p=5.48x10^-07  Sex*Genotype p=0.21743  Tukey post hoc tests:  Ctrl vs *bnlRNAi* Males p=0.0082902  Ctrl vs *bnlRNAi* Females p=0.0000554  No. DSRF-positive nuclei  ANOVA: No. DSRF~sex*genotype  Sex p=6.26x10^-14  Genotype p=2.91x10^-16  Sex*Genotype p=0.000105  Tukey post hoc tests:  Ctrl vs *bnlRNAi* Males p=0.0008435  Ctrl vs *bnlRNAi* Females p<2x10^-16 |
| 2i | *Hand > Ctrl* Male  *Hand > bnlRNAi* Male  *Hand > Ctrl* Female  *Hand > bnlRNAi* Female | 38  47  40  33 |  |
| 2j-k | *Hand > Ctrl* Male  *Hand > bnlRNAi* Male  *Hand > Ctrl* Female  *Hand > bnlRNAi* Female | 38  47  40  33 | MDS stats – see Methods  Ctrl vs *bnlRNAi* Males p=5.22x10^-7  Ctrl vs *bnlRNAi* Females p=0.0024 |
| 3a | *bnl-lexA > myr::GFP*  Male 5hAPE  *bnl-lexA > myr::GFP* Female 5hAPE  *bnl-lexA > myr::GFP*  Male 7daysAPE  *bnl-lexA > myr::GFP* Female 7daysAPE | 27  28  35  37 |  |
| 3b | *w, Su(H)GBE-LacZ/ w; esg-Gal4 NP7397, UAS-GFP, Tub-Gal80TS /+; +/ +* | 30 x 3 | Hudry et al 2016 |
| 3c | *OregonR* Male  *OregonR* Female | 36  34 | No. DSRF-positive nuclei  Two-sample t-test: p=2.264x10^-8 |
| 3d | *btl > myr::GFP* Male  *btl > myr::GFP* Female | 20  16 | Two-sample t-tests:  Total tracheal length p=3.086x10^-14  Trachea coverage p=0.0007972  Mean branch length p=0.02804  No. of sholl intersections p=7.117x10^-12 |
| 4b | *Hand > Ctrl* Male  *Hand > SxlRNAi* Male  *Hand > Ctrl* Female  *Hand > SxlRNAi* Female | 14  19  19  17 |  |
| 4c | *Hand > Ctrl* Male  *Hand > SxlRNAi* Male  *Hand > Ctrl* Female  *Hand > SxlRNAi* Female | 21  20  20  19 | No. DSRF-positive nuclei  ANOVA: No. DSRF~sex*genotype  Sex p= 1.91x10^-09  Genotype p= 0.00462  Sex*Genotype p= 4.60x10^-07  Tukey post hoc tests:  Ctrl vs *SxlRNAi* Males p=0.2998178  Ctrl vs *SxlRNAi* Females p=0.4x10^-6 |
| 4d | *Hand > Ctrl* Male  *Hand > SxlRNAi* Male  *Hand > Ctrl* Female  *Hand > SxlRNAi* Female | 41  24  44  31 | (Procrustes ANOVA results in Supplementary Tables 34-35) |
| 4e-f | *Hand > Ctrl* Male  *Hand > SxlRNAi* Male  *Hand > Ctrl* Female  *Hand > SxlRNAi* Female | 41  24  44  31 | MDS stats – see Methods  Ctrl vs *SxlRNAi* Males p=0.30  Ctrl vs *SxlRNAi* Females  p=5.7*10^-6 |
| 4h | *Hand > Ctrl* Male  *Hand > SxlOE* Male  *Hand > Ctrl* Female  *Hand > SxlOE* Female | 18  9  17  15 |  |
| 4i | *Hand > Ctrl* Male  *Hand > SxlOE* Male  *Hand > Ctrl* Female  *Hand > SxlOE* Female | 20  18  19  20 | No. DSRF-positive nuclei  ANOVA: No. DSRF~sex*genotype  Sex p<2.16x10^-16  Genotype p =8.33x10^-06  Sex*Genotype p=0.000899  Tukey post hoc tests:  Ctrl vs *SxlOE* Males p=0.8x10^-6  Ctrl vs *SxlOE* Females p=0.7590367 |
| 4j | *Hand > Ctrl* Male  *Hand > SxlOE* Male  *Hand > Ctrl* Female  *Hand > SxlOE* Female | 29  30  19  13 | (Procrustes ANOVA results in Supplementary Tables 36-37) |
| 4k-l | *Hand > Ctrl* Male  *Hand > SxlOE* Male  *Hand > Ctrl* Female  *Hand > SxlOE* Female | 29  30  19  13 | MDS stats – see Methods  Ctrl vs *SxlRNAi* Males p=0.0012  Ctrl vs *SxlRNAi* Females p=0.84 |
| 5b | *ubi-ODD-GFP, ubi-RFP*  *Hand > Ctrl* Male  *Hand > bnlRNAi* Male  *Hand > Ctrl* Female  *Hand > bnlRNAi* Female  *Ldh::GFP*  *Hand > Ctrl* Male  *Hand > bnlRNAi* Male  *Hand > Ctrl* Female  *Hand > bnlRNAi* Female | 19  26  24  20  28  19  20  21 |  |
| 5c | *Hand > Ctrl* Male  *bnlRNAi Ctrl* Male  *Hand > bnlRNAi* Male  *Hand > Ctrl* Female  *bnlRNAi Ctrl* Female  *Hand > bnlRNAi* Female | 20  19  22  17  18  13 | No. pH3-positive cells  ANOVA: No. pH3~sex*genotype  Sex p=4.66x10^-14  Genotype p=0.000140  Sex*Genotype p=0.000162  Tukey post hoc tests:  Gal4 Ctrl vs *bnlRNAi* Males p=0.9933645  UAS Ctrl vs *bnlRNAi* Males p=0.9957703  Gal4 Ctrl vs *bnlRNAi* Females p=0.0000577  UAS Ctrl vs *bnlRNAi* Females p=0.0000007 |
| 5d | *Hand > Ctrl*  5% Suc Male  *bnlRNAi Ctrl*  5% Suc Male  *Hand > bnlRNAi*  5% Suc Male  *Hand > Ctrl*  5% Suc+3%DSS Male  *bnlRNAi Ctrl*  5% Suc+3%DSS Male  *Hand > bnlRNAi*  5% Suc+3%DSS Male  *Hand > Ctrl*  5% Suc Female  *bnlRNAi Ctrl*  5% Suc Female  *Hand > bnlRNAi*  5% Suc Female  *Hand > Ctrl*  5% Suc+3%DSS Female  *bnlRNAi Ctrl*  5% Suc+3%DSS Female  *Hand > bnlRNAi*  5% Suc+3%DSS Female | 12  26  12  9  22  21  20  23  22  34  20  19 | No. pH3-positive cells  ANOVA: No. pH3~sex*genotype*treatment*batch  Sex p=6.39x10^-12  Treatment p=4.83x10^-13  Genotype p=0.47640  Batch p=0.00965  Sex*Treatment p=0.00320  Genotype*Treatment p=0.00317  Tukey post hoc tests:  Gal4 Ctrl Suc vs Gal4 Ctrl DSS Males p=0.058204  UAS Ctrl Suc vs UAS Ctrl DSS Males p=0.995511  bnlRNAi Suc vs bnlRNAi DSS Males p=0.954353  Gal4 Ctrl Suc vs Gal4 Ctrl DSS Females p=3.63x10^-09  UAS Ctrl Suc vs UAS Ctrl DSS Females p=0.01052  bnlRNAi Suc vs bnlRNAi DSS Females p=0.266066 |
| 5e | *Hand > Ctrl* Female  *bnlRNAi Ctrl* Female  *Hand > bnlRNAi* Female | 13  10  14 | No. Eggs/fly/day  ANOVA: No. eggs~day*genotype  Day p<2x10^-16  Genotype p=1.12x10^-15  Day*Genotype p=0.0028  Tukey post hoc tests:  Gal4 Ctrl vs *bnlRNAi* day2 p<2x10^-16  UAS Ctrl vs *bnlRNAi* day2 p<2x10^-16  Gal4 Ctrl vs *bnlRNAi* day5 p=0.0152637  UAS Ctrl vs *bnlRNAi* day5 p=0.0010075  Gal4 Ctrl vs *bnlRNAi* day8 p=0.7251289  UAS Ctrl vs *bnlRNAi* day8 p=0.0005415 |
| 5f | *Hand > Ctrl* Female  *SxlRNAi Ctrl* Female  *Hand > SxlRNAi* Female | 18  18  19 | No. Eggs/fly/day  ANOVA: No. eggs~day*genotype  Day p< 2x10^-16  Genotype p=2.48x10^-12  Day*Genotype p=0.00387  Tukey post hoc tests:  Gal4 Ctrl vs Sxl*RNAi* day2 p=0.0000303  UAS Ctrl vs Sxl*RNAi* day2 p<2x10^-16  Gal4 Ctrl vs Sxl*RNAi* day5 p=0.1367759  UAS Ctrl vs Sxl*RNAi* day5 p=0.0013994  Gal4 Ctrl vs Sxl*RNAi* day8 p=0.9894002  UAS Ctrl vs Sxl*RNAi* day8 p=0.5370729 |
| ED1c | *OregonR* Male  *OregonR* Female | 62  60 | (Procrustes ANOVA results in Supplementary Tables 5-6) |
| ED1d-e | *OregonR* Male  *OregonR* Female | 44  40 | Relative length  ANOVA: Length~region*sex  Region p<2x10^-16  Sex p=1  Region*Sex p<2x10^-16  Tukey post hoc tests:  Anterior midgut male vs female p<2x10^-16  Midgut coils male vs female p<2x10^-16  Hindgut male vs female p<2x10^-16  Absolute length  ANOVA: Length~region*sex  Region p<2x10^-16  Sex p<2x10^-16  Region*Sex p<2x10^-16  Tukey post hoc tests:  Whole gut male vs female p<2x10^-16  Anterior midgut male vs female p=0.98495  Midgut coils male vs female p<2x10^-16  Hindgut male vs female p=0.07052 |
| ED1f-h | *OregonR* Male  *OregonR* Female | 41  39 | Radius  Two-sample t-test: p=2.794x10^-11 |
| ED1i-k | *CantonS* Male  *CantonS* Female | 41  27 | (Procrustes ANOVA results in Supplementary Tables 7-8)  Gut length  Two-sample t-test: p= p<2.2*10^-16 |
| ED1l-n | *w^1118^ Male*  *w^1118^ Female* | 38  40 | (Procrustes ANOVA results in Supplementary Tables 9-10)  Gut length:  Two-sample t-test: p= p<2.2*10^-16 |
| ED1p-r | *OregonR* Male  *OregonR* Female  *ovo^D1^* Female | 62  60  28 | (Procrustes ANOVA results in Supplementary Table 11)  Gut length  ANOVA: gut length~group  Group p<2x10^-16  Tukey post hoc tests:  *OR* Male vs *OR* Female p<2x10^-16  *OR* Male vs *ovo^D1^* p<2x10^-16  *OR* Female vs *ovo^D1^* p=0.0001182 |
| ED1t-v | *OregonR ad libitum*  Male  *OregonR starved 48h* Male  *OregonR ad libitum* Female  *OregonR starved 48h* Female | 18  25  22  16 | (Procrustes ANOVA results in Supplementary Tables 12-13)  Gut length  ANOVA: gut length~sex*genotype  Sex p<2x10^-16  Genotype p=1.69*10^-4  Sex*Genotype p=2.64*10^-4  Tukey post hoc tests:  Ctrl Males vs Starved Males p=0.9938  Ctrl Females vs Starved Females p=2.9*10^-6 |
| ED2a-c | *OregonR* Male | 44 |  |
| ED2d | *OregonR* Female | 44 |  |
| ED2e-f | *OregonR* Male  *OregonR* Female | 44  44 |  |
| ED2g | *OregonR* Male | 44 |  |
| ED2h | *OregonR* Female | 44 |  |
| ED2i | *OregonR* Male | 62 | Testes Volume  Paired t-test: p=0.1214 |
| ED2j | *OregonR* Female  *ovo^D1^* Female | 43  28 |  |
| ED2k | *OregonR* Female  *ovo^D1^* Female | 60  28 | Ovary Volume  Two-sample t-test: p<2.2*10^-16 |
| ED2l | *OregonR* Female  *ovo^D1^* Female | 43  29 | Gut Volume  Two-sample t-test: p=1.281*10^-5 |
| ED2m | *OregonR* control Female  *OregonR* 48h starved Female | 22  16 |  |
| ED2n | *OregonR* control Female  *OregonR* 48h starved Female | 22  16 | Ovary Volume  Two-sample t-test: p=2.048*10^-8 |
| ED2o | *OregonR* control Female  *OregonR* 48h starved Female | 22  16 | Gut Volume  Two-sample t-test: p=0.3166 |
| ED2p | *OregonR* Male  *OregonR* Female | 62  60 | Gut Length  Two-sample t-test: p<2.2x10^-16 |
| ED2q | *OregonR* Male  *OregonR* Female | 62  60 | Gonad Volume  Two-sample t-test: p<2.2x10^-16 |
| ED2r | *OregonR* Male  *OregonR* Female | 62  60 | Crop Volume  Two-sample t-test: p=0.9452 |
| ED3a-c | *btl > myrGFP* Female  *OregonR* Female  *btl > myrGFP* Male  *OregonR* Male | 21  40  25  44 |  |
| ED3d | *trh*> ctrl Male  *trh* > *Bax* Male  *trh* > ctrl Female  *trh* > *Bax* Female | 29  35  14  41 | (Procrustes ANOVA results in Supplementary Tables 20-21)  Gut length  ANOVA: gut length~sex*genotype  Sex p=1.79*10^-15  Genotype p=1.88*10^-11  Sex*Genotype p=8.53*10^-5  Tukey post hoc tests:  Ctrl vs *Bax* Males p=0.008537  Ctrl vs *Bax* Females p<2x10^-16 |
| ED3e | *Hand > Ctrl* Male  *Hand > bnlRNAi* Male  *Hand > Ctrl* Female  *Hand > bnlRNAi* Female | 38  47  40  33 | (Procrustes ANOVA results in Supplementary Tables 24-25)  Gut length  ANOVA: gut length~sex*genotype  Sex p<2x10^-16  Genotype p=4.98*10^-4  Sex*Genotype p=0.1202  Tukey post hoc tests:  Ctrl vs *bnlRNAi* Males p=0.41302  Ctrl vs *bnlRNAi* Females p=0.00270 |
| ED3f | *Hand^ts^ > Ctrl* Male  *Hand^ts^ > bnlRNAi* Male  *Hand^ts^ > Ctrl* Female  *Hand^ts^ > bnlRNAi* Female | 19  12  14  16 | Total tracheal length  ANOVA: total tracheal length~sex*genotype  Sex p=3.43x10^-05  Genotype p<2.16x10-16  Sex*Genotype p=0.000586  Tukey post hoc tests:  Ctrl vs *bnlRNAi* Males p<2.16x10-16  Ctrl vs *bnlRNAi* Females p<2.16x10-16 |
| ED3h | *vm > Ctrl Male*  *vm > bnlRNAi Male*  *vm > Ctrl Female*  *vm > bnlRNAi Female* | 25  20  20  24 | No. DSRF-positive nuclei  ANOVA: No. DSRF~sex*genotype  Sex p=9.37x10^-09  Genotype p<2.16x10-16  Sex*Genotype p=0.151  Tukey post hoc tests:  Ctrl vs *bnlRNAi* Males p<2.16x10-16  Ctrl vs *bnlRNAi* Females p<2.16x10-16 |
| ED3i-k | *vm > Ctrl Male*  *vm > bnlRNAi Male*  *vm > Ctrl Female*  *vm > bnlRNAi Female* | 20  22  21  21 | (Procrustes ANOVA results in Supplementary Tables 26-27)  Gut length  ANOVA: gut length~sex*genotype  Sex p<2x10^-16  Genotype p=2.72*10^-3  Sex*Genotype p=0.6168  Tukey post hoc tests:  Ctrl vs *bnlRNAi* Males p=0.0610  Ctrl vs *bnlRNAi* Females p=0.2650 |
| ED4a | *bnl-Gal4 > Stinger::GFP* Male 5hAPE  *bnl-Gal4 > Stinger::GFP* Female 5hAPE  *bnl-Gal4 > Stinger::GFP* Male 7daysAPE  *bnl-Gal4 > Stinger::GFP* Female 7daysAPE | 16  13  14  14 |  |
| ED4b | *bnl-lexA > myr::GFP*  Male 5hAPE  *OregonR*  Male  *bnl-lexA > myr::GFP* Female 5hAPE  *OregonR*  Female | 27  44  28  40 |  |
| ED5b | *QF6>mtdTomato*  *mex1> Ctrl* Male  *mex1> bnlRNAi* Male  *mex1> Ctrl* Female  *mex1> bnlRNAi* Female  DSRF staining  *mex1 > ctrl* Male  *mex1 > bnlRNAi* Male  *mex1> ctrl* Female  *mex1> bnlRNAi* Female | 41  53  26  28  28  23  26  20 | Total tracheal length:  ANOVA: total tracheal length~sex*genotype  Sex p<2.16x10-16  Genotype p=0.610  Sex*Genotype p=0.873  Tukey post hoc tests:  Ctrl vs *bnlRNAi* Males p=0.9581034  Ctrl vs *bnlRNAi* Females p=0.9978293  No DSRF-positive nuclei  ANOVA: No DSRF~sex*genotype  Sex p<2.16x10-16  Genotype p=0.263  Sex*Genotype p=0.562  Tukey post hoc tests:  Ctrl vs *bnlRNAi* Males p=0.6169712  Ctrl vs *bnlRNAi* Females p=0.9850769 |
| ED5d | *QF6>mtdTomato*  *mex1, esg^ts^> Ctrl*  Male  *mex1, esg^ts^> btlRNAi* Male  *mex1, esg^ts^> Ctrl*  Female  *mex1, esg^ts^> btlRNAi,* Female  DSRF staining  *mex1, esg^ts^> Ctrl*  Male  *mex1, esg^ts^> btlRNAi* Male  *mex1, esg^ts^> Ctrl*  Female  *mex1, esg^ts^> btlRNAi,* Female | 22  19  21  24  22  18  22  14 | Total tracheal length  ANOVA: total tracheal length~sex*genotype  Sex p<2.16x10^-16  Genotype p=0.200  Sex*Genotype p=0.135  Tukey post hoc tests:  Ctrl vs btlRNAi Males p=0.9971781  Ctrl vs btlRNAi Females p=0.2050567  No. DSRF-positive nuclei  ANOVA: No. DSRF~sex*genotype  Sex p<2.16x10^-16  Genotype p=0.122  Sex*Genotype p=0.695  Tukey post hoc tests:  Ctrl vs btlRNAi Males p=0.4942157  Ctrl vs btlRNAi Females p=0.8642681 |
| ED5e-f | *mex1 > Ctrl* Male  *mex1 > bnlRNAi* Male  *mex1> Ctrl* Female  *mex1> bnlRNAi* Female | 28  23  15  31 | (Procrustes ANOVA results in Supplementary Tables 22-23) |
| ED5g-h | *mex1, esg^ts^> Ctrl*  Male  *mex1, esg^ts^> btlRNAi* Male  *mex1, esg^ts^> Ctrl*  Female  *mex1, esg^ts^> btlRNAi* Female | 30  38  31  33 | (Procrustes ANOVA results in Supplementary Tables 28-29) |
| ED6a | *w^1118^* Male  *w^1118^* Female  *btl > StingerGFP* Male  *btl > StingerGFP* Female | 28  23  16  20 | No. DSRF-positive nuclei  Two-sample t-test:  *w^1118^* Male vs Female p=4.983x10^-8  No. GFP-positive nuclei  Two-sample t-test:  *btl > StingerGFP* Male vs Female p=3.131x10^-15 |
| ED6b | *QF6 > mtdTomato* Male  *QF6> mtdTomato*Female  *DSRF > CD8::GFP* Male  *DSRF > CD8::GFP* Female  *trh > CD8::GFP* Male  *trh > CD8::GFP* Female | 17  17  25  28  33  33 | Total trachea length  Two-sample t-tests:  *QF6 > mtdTomato* Male vs Female p=0.0004375  *DSRF > CD8::GFP* Male vs Female p=8.175x10^-10  *trh > CD8::GFP* Male vs Female p<2.2x10^-16  Mean branch length  Two-sample t-tests:  *QF6 > mtdTomato* Male vs Female p=0.6379  *DSRF > CD8::GFP* Male vs Female p=0.0003203  *trh > CD8::GFP* Male vs Female p=2.592x10^-10  No. Sholl intersections  Two-sample t-tests:  *QF6 > mtdTomato* Male vs Female p 0.00363  *DSRF > CD8::GFP* Male vs Female p=1.51x10^-12  *trh > CD8::GFP* Male vs Female p<2.2x10^-16 |
| ED7b | *btl >myr::GFP*  *btl > Ctrl* Male  *btl > traRNAi* Male  *btl > Ctrl* Female  *btl > traRNAi* Female  DSRF staining  *btl > Ctrl* Male  *btl > traRNAi* Male  *btl > Ctrl* Female  *btl > traRNAi* Female | 19  25  21  18  19  25  20  18 | Total tracheal length  ANOVA: total tracheal length~sex*genotype  Sex p=2.92x10^-11  Genotype p=0.4156  Sex*Genotype p=0.00311  Tukey post hoc tests:  Ctrl vs *traRNAi* Males p=0.0422180  Ctrl vs *traRNAi* Females p=0.3563761  No. DSRF-positive nuclei  ANOVA: No. DSRF~sex*genotype  Sex p<2x10^-16  Genotype p=1.71*10^-4  Sex*Genotype p=1.008*10^-3  Tukey post hoc tests:  Ctrl vs *traRNAi* Males p=0.94733  Ctrl vs *traRNAi* Females p=0.0000094 |
| ED7d | *btl >myr::GFP*  *btl > Ctrl* Male  *btl > traRNAi* Male  *btl > Ctrl* Female  *btl > traRNAi* Female  DSRF staining  *btl > Ctrl* Male  *btl > traRNAi* Male  *btl > Ctrl* Female  *btl > traRNAi* Female | 23  19  21  18  23  19  22  18 | Total tracheal length  ANOVA: total tracheal length~sex*genotype  Sex p<2x10^-16  Genotype p=1.65*10^-8  Sex*Genotype p=0.0432  Tukey post hoc tests:  Ctrl vs *traRNAi* Males p=0.01367  Ctrl vs *traRNAi* Females p=0.0000006  No. DSRF-positive nuclei  ANOVA: No. DSRF~sex*genotype  Sex p<2x10^-16  Genotype p=0.3379  Sex*Genotype p=0.0112  Tukey post hoc tests:  Ctrl vs *traRNAi* Males p=0.67552  Ctrl vs *traRNAi* Females p=0.06251 |
| ED7e-f | *btl > Ctrl* Male  *btl > traRNAi* Male  *btl > Ctrl* Female  *btl > traRNAi* Female | 19  14  12  15 | (Procrustes ANOVA results in Supplementary Tables 30-31) |
| ED7g | *btl > Ctrl* Male  *btl > traRNAi* Male  *btl > Ctrl* Female  *btl > traRNAi* Female | 19  14  12  15 | Gut length  ANOVA: gut length~sex*genotype  Sex p<2x10^-16  Genotype p=0.619  Sex*Genotype p=0.786  Tukey post hoc tests:  Ctrl vs *traRNAi* Males p=0.94530  Ctrl vs *traRNAi* Females p=0.99911 |
| ED8b | *Hand > Ctrl* Male  *Hand > traRNAi* Male  *Hand > Ctrl* Female  *Hand > traRNAi* Female | 17  18  15  17 |  |
| ED8c | *QF6>mtdTomato*  *Hand > Ctrl* Male  *Hand > traRNAi* Male  *Hand > Ctrl* Female  *Hand > traRNAi* Female  DSRF staining  *Hand > Ctrl* Male  *Hand > traRNAi* Male  *Hand > Ctrl* Female  *Hand > traRNAi* Female | 25  17  20  30  23  26  24  24 | Total tracheal length  ANOVA: total tracheal length~sex*genotype  Sex p=5.94^-16  Genotype p=0.0139  Sex*Genotype p=0.2051  Tukey post hoc tests:  Ctrl vs *traRNAi* Males p=0.87312  Ctrl vs *traRNAi* Females p=0.03940  No. DSRF-positive nuclei  ANOVA: No. DSRF~sex*genotype  Sex p=1.48*10^-13  Genotype p=2.27*10^-11  Sex*Genotype p=3.73*10^-10  Tukey post hoc tests:  Ctrl vs *traRNAi* Males p=0.96698  Ctrl vs *traRNAi* Females p<2x10^-16 |
| ED8d-g | *Hand > Ctrl* Male  *Hand > traRNAi* Male  *Hand > Ctrl* Female  *Hand > traRNAi* Female | 31  31  28  31 | (Procrustes ANOVA results in Supplementary Tables 32-33)  MDS stats – see Methods  Ctrl vs *traRNAi* Males p=0.058  Ctrl vs *traRNAi* Females p=0.0025 |
| ED8h-i | *Hand > Ctrl* Male  *Hand > SxlRNAi* Male  *Hand > Ctrl* Female  *Hand > SxlRNAi* Female | 41  24  44  31 | (Procrustes ANOVA results in Supplementary Tables 34-35)  Gut length  ANOVA: gut length~sex*genotype  Sex p<2x10^-16  Genotype p=2.48*10^-5  Sex*Genotype p=1.58*10^-7  Tukey post hoc tests:  Ctrl vs *SxlRNAi* Males p=0.6639  Ctrl vs *SxlRNAi* Females p<2x10^-16 |
| ED8j-k | *Hand > Ctrl* Male  *Hand > Sxl* Male  *Hand > Ctrl* Female  *Hand > Sxl* Female | 29  30  19  20 | (Procrustes ANOVA results in Supplementary Tables 36-37)  Gut length  ANOVA: gut length~sex*genotype  Sex p<2x10^-16  Genotype p=0.2214  Sex*Genotype p=0.0329  Tukey post hoc tests:  Ctrl vs *Sxl OE* Males p=0.1133  Ctrl vs *Sxl OE* Females p=0.7295 |
| ED10a | *Hand > Ctrl* Male  *bnlRNAi Ctrl* Male  *Hand > bnlRNAi* Male  *Hand > Ctrl* Female  *bnlRNAi Ctrl* Female  *Hand > bnlRNAi* Female | 25  25  28  18  18  20 | No. pH3-positive cells  ANOVA: No. pH3~sex*genotype  Sex p=1.73x10^-08  Genotype p=0.2087  Sex*Genotype p=0.0442  Tukey post hoc tests:  Gal4 Ctrl vs *bnlRNAi* Males p=0.9999999  UAS Ctrl vs *bnlRNAi* Males p=0.9946773  Gal4 Ctrl vs *bnlRNAi* Females p=0.1552679  UAS Ctrl vs *bnlRNAi* Females p=0.9972897 |
| ED10b | *Hand > Ctrl* Male  *bnlRNAi Ctrl* Male  *Hand > bnlRNAi* Male  *Hand > Ctrl* Female  *bnlRNAi Ctrl* Female  *Hand > bnlRNAi* Female | 28  25  31  27  19  44 | No. sips  ANOVA: No. sips~sex*genotype  Sex p=5.43x10^-12  Genotype p=0.0326  Sex*Genotype p=0.9309  Tukey post hoc tests:  Gal4 Ctrl vs *bnlRNAi* Malesp=0.3652270  UAS Ctrl vs *bnlRNAi* Males p=0.6837912  Gal4 Ctrl vs *bnlRNAi* Females p=0.6304898  UAS Ctrl vs *bnlRNAi* Females p=0.8594568 |
| ED10c-d | *Hand > Ctrl* Male  *Hand > bnlRNAi* Male  *Hand > Ctrl* Female  *Hand > bnlRNAi* Female | 8x3  8x3  8x3  8x3 | Blue dye absorbance (ad libitum)  ANOVA: Dye absorbance~sex*genotype  Sex p=5.52x10^-08  Genotype p=0.821  Sex*Genotype p=0.317  Tukey post hoc tests:  Ctrl vs *bnlRNAi* Males p=0.9434651  Ctrl vs *bnlRNAi* Females p=0.8142331  Blue dye absorbance (starved-refed)  ANOVA: Dye absorbance ~sex*genotype  Sex p=0.135  Genotype p=0.253  Sex*Genotype p=0.991  Tukey post hoc tests:  Ctrl vs *bnlRNAi* Males p=0.8379774  Ctrl vs *bnlRNAi* Females p=0.8459318 |
| ED10e | *Hand > Ctrl* Male  *bnlRNAi Ctrl* Male  *Hand > bnlRNAi* Male  *Hand > Ctrl* Female  *bnlRNAi Ctrl* Female  *Hand > bnlRNAi* Female | 73  73  42  71  67  36 | Logistic regression:  Transit~genotype+sex  Genotype p =0.148  Sex p=1.25x10^-06 |
| ED10f | *Hand > Ctrl* Male  *Hand > bnlRNAi* Male  *Hand > Ctrl* Female  *Hand > bnlRNAi* Female | 10x6  10x6  10x6  10x6 | No. Faecal deposits  ANOVA: No. deposits~sex*genotype  Sex p=4.02x10^-13  Genotype p=0.0444  Sex*Genotype p=0.8159  Tukey post hoc tests:  Ctrl vs *bnlRNAi* Males p=0.3702838  Ctrl vs *bnlRNAi* Females p=0.5642974 |
| ED10 g-h | *Hand > Ctrl* Male  *bnlRNAi Ctrl* Male  *Hand > bnlRNAi* Male  *Hand > Ctrl* Female  *bnlRNAi Ctrl* Female  *Hand > bnlRNAi* Female | 82  86  79  88  84  85 | Log-rank (Mantel-Cox) test  Gal4 Ctrl vs *bnlRNAi* Males p=0.0163  UAS Ctrl vs *bnlRNAi* Males p<2.16x10^-16  Gal4 Ctrl vs *bnlRNAi* Females p=0.0077  UAS Ctrl vs *bnlRNAi* Females p<2.16x10^-16 |

**3. Organ contact frequencies**

**Crop and ovary contact in female *OregonR* flies:**

| Crop contact with | Percentage of flies |
| --- | --- |
| Left ovary | 46.9 |
| Right ovary | 6.3 |
| Both ovaries | 4.7 |
| Neither ovary | 42.2 |

**Crop duct configuration joining gut to crop in male and female *OregonR* flies:**

| Configuration | Percentage of males | Percentage of females |
| --- | --- | --- |
| S-shape | 34.9 | 37.2 |
| Diagonal straight | 55.8 | 41.9 |
| One-sided | 4.7 | 7 |
| Inverted | 4.7 | 14 |
