# Supplementary material for: The sex of organ geometry
Source: Nature. 2024 May 29;630(8016):392–400. doi: 10.1038/s41586-024-07463-4 (PMC11168936; doi:10.1038/s41586-024-07463-4)
Supplement: Supplementary file 2 — Reporting Summary [file 41586_2024_7463_MOESM2_ESM.pdf]

nature portfolio

Corresponding author(s): Irene Miguel-Aliaga

Last updated by author(s): Apr 11, 2024

Reporting Summary

Nature Portfolio wishes to improve the reproducibility of the work that we publish. This form provides structure for consistency and transparency in reporting. For further information on Nature Portfolio policies, see our Editorial Policies and the Editorial Policy Checklist.

Statistics

For all statistical analyses, confirm that the following items are present in the figure legend, table legend, main text, or Methods section.

n/a	Confirmed	
☐	☒	The exact sample size (n) for each experimental group/condition, given as a discrete number and unit of measurement
☐	☒	A statement on whether measurements were taken from distinct samples or whether the same sample was measured repeatedly
☐	☒	The statistical test(s) used AND whether they are one- or two-sided<br>*Only common tests should be described solely by name; describe more complex techniques in the Methods section.*
☐	☒	A description of all covariates tested
☐	☒	A description of any assumptions or corrections, such as tests of normality and adjustment for multiple comparisons
☐	☒	A full description of the statistical parameters including central tendency (e.g. means) or other basic estimates (e.g. regression coefficient) AND variation (e.g. standard deviation) or associated estimates of uncertainty (e.g. confidence intervals)
☐	☒	For null hypothesis testing, the test statistic (e.g. F, t, r) with confidence intervals, effect sizes, degrees of freedom and P value noted<br>*Give P values as exact values whenever suitable.*
☒	☐	For Bayesian analysis, information on the choice of priors and Markov chain Monte Carlo settings
☒	☐	For hierarchical and complex designs, identification of the appropriate level for tests and full reporting of outcomes
☐	☒	Estimates of effect sizes (e.g. Cohen's d, Pearson's r), indicating how they were calculated

Our web collection on statistics for biologists contains articles on many of the points above.

Software and code

Policy information about availability of computer code

Data collection	Zeiss Xradia Versa 510, Bruker Skyscan 1272 and Bruker Skyscan 1172 for microCT image acquistion; Zeiss Reconstructor v11 and Bruker Nrecon v2 for microCT image reconstruction; Leica SP5 and Leica TCS SP8 DLS for confocal image acquisition; Nikon CSU-W1 SoRa spinning disk microscope with NIS-Elements software for laser ablation experiments; FlyPAD device with Bonsai software for feeding assays.
Data analysis	Imaris x64 v9.9.0 with 'Filament tracer' and 'Batch' packages for confocal image analysis; FIJI v2.0.0-rc-69/1.52p for confocal image and microCT scan analysis with Simple neurite tracer plugin v3.1.6 for centreline tracing; R v3.6.0 with packages 'geomorph' v3.2.1, 'nat' v1.8.18, 'RRPP' v0.5.2 for geometric morphometric analysis of gut shape, and 'RANN' v1.8.18 for proximity analysis; R v4.2.1 with packages 'ggplot2' v3.4.0, R 'dplyr' v1.0.10 for data visualisation and statistical analysis and package 'VGAM' v1.1 for intestinal transit; Adobe Photoshop v25.3.1 for tracheal coverage; ITK-snap v3.8.0 for microCT segmentation; Meshlab v2020.07, Paraview 5.10.0 for 3D mesh analysis; Mathematica v13.1, Python v3.10.0 with packages 'NUMPY' and 'scikit-fda' for gut shape analysis; MATLAB R2023b for analysis of FlyPAD recordings; T.U.R.D software for intestinal excretion; GraphPad Prism v9.4.1 for starvation resistance. Custom code is available on GitHub via Zenodo: http://doi.org/10.5281/zenodo.10905446

For manuscripts utilizing custom algorithms or software that are central to the research but not yet described in published literature, software must be made available to editors and reviewers. We strongly encourage code deposition in a community repository (e.g. GitHub). See the Nature Portfolio guidelines for submitting code & software for further information.

Data

Policy information about availability of data

All manuscripts must include a data availability statement. This statement should provide the following information, where applicable:

- Accession codes, unique identifiers, or web links for publicly available datasets
- A description of any restrictions on data availability
- For clinical datasets or third party data, please ensure that the statement adheres to our policy

All reconstructed microCT scans, gut centreline files and organ segmentation files are available on Figshare with the identifier: https://doi.org/10.25418/crick.25598859. All remaining data generated or analysed during this study are included in this published article (and its Extended Data/Supplementary Information files) and accompanying source data files. Further information can be requested from the corresponding author.

Research involving human participants, their data, or biological material

Policy information about studies with human participants or human data. See also policy information about sex, gender (identity/presentation), and sexual orientation and race, ethnicity and racism.

Reporting on sex and gender	N/A
Reporting on race, ethnicity, or other socially relevant groupings	N/A
Population characteristics	N/A
Recruitment	N/A
Ethics oversight	N/A

Note that full information on the approval of the study protocol must also be provided in the manuscript.

Field-specific reporting

Please select the one below that is the best fit for your research. If you are not sure, read the appropriate sections before making your selection.

☒ Life sciences ☐ Behavioural & social sciences ☐ Ecological, evolutionary & environmental sciences

For a reference copy of the document with all sections, see nature.com/documents/nr-reporting-summary-flat.pdf

Life sciences study design

All studies must disclose on these points even when the disclosure is negative.

Sample size	Sample sizes are provided for each experiment in Supplementary Information. Fly numbers are not limiting so no power calculations were used to pre-determine sample size. Oversampling was mitigated by choosing sample sizes based on previous knowledge of phenotypic variability in controls and other mutants. In particular, we used similar sample sizes used previously to determine variability in Drosophila gut length, as reported in Hudry et al 2019 (https://doi.org/10.1038/nature16953), White et al. 2021 (https://doi.org/10.1073/pnas.2018112118) and Bonfini et al. 2021 (https://doi.org/10.7554/eLife.64125). Similar sample sizes for different animal groups (e.g.mutants vs controls) were tested in the same experimental design.
Data exclusions	All measured datapoints are displayed in figures and outliers were not excluded from data analysis
Replication	Experiments were typically replicated 3 times and only those experiments for which repeats gave comparable outcomes are included in the manuscript.
Randomization	Experimental and control flies were bred in identical conditions, and were randomised whenever possible (for example, with regard to housing, position in tray). Control and experimental samples were dissected and processed at the same time and on the same slides or tips for confocal imaging and microCT. Experiments were controlled for sex, mating status, genotype and age. Detailed information is provided in the main text and, more systematically for each figure panel and in the Supplementary Information.
Blinding	Blinding was performed for a subset of experiments. Quantification of DSRF stainings, filament tracing of QF6>tomato-labelled trachea and quantifications of bnl expression along gut length was done on data blinded for genotype. Blinding for sex was not possible as this is visually obvious by differences in the length and diameter of the Drosophila gut. Similarly blinding for sex was not possible for microCT scans as ovaries and testes were visible in the images.

Reporting for specific materials, systems and methods

We require information from authors about some types of materials, experimental systems and methods used in many studies. Here, indicate whether each material, system or method listed is relevant to your study. If you are not sure if a list item applies to your research, read the appropriate section before selecting a response.

Materials & experimental systems

n/a	Involved in the study	
☐	☒	Antibodies
☒	☐	Eukaryotic cell lines
☒	☐	Palaeontology and archaeology
☐	☒	Animals and other organisms
☒	☐	Clinical data
☒	☐	Dual use research of concern
☒	☐	Plants

Methods

n/a	Involved in the study	
☒	☐	ChIP-seq
☒	☐	Flow cytometry
☒	☐	MRI-based neuroimaging

Antibodies

Antibodies used

Primary antibodies: Mouse anti-DSRF (Active Motif, 39093), goat anti-GFP (Abcam, ab5450), rabbit anti-mCherry (Abcam, ab167453), mouse anti-prospero (DSHB, MR1A) and anti-horseradish peroxidase (HRP) Rhodamine (TRITC)-conjugated (Jackson ImmunoResearch, 123-025-021). Secondary antibodies: anti-rabbit FITC-conjugated (Jackson ImmunoResearch, 711-97-003), anti-mouse Cy3-conjugated (Jackson ImmunoResearch715-166-150), anti-mouse Cy5-conjugated (Jackson ImmunoResearch, 715-175-151) and anti-goat FITC-conjugated (Jackson ImmunoResearch, 112-095-044).

Validation

All antibodies used were previously published and tested in Drosophila melanogaster tissues. Working concentrations were based on previously published assessments: Mouse anti-DSRF (Active Motif, 39093) was tested and reported to label terminal tracheal cells in the Drosophila gut in Linneweber et al. 2014 (https://doi.org/10.1016/j.cell.2013.12.008); Mouse anti-prospero (DSHB, MR1A) was shown to label Drosophila gut enteroendocrine cells in Michelli and Perrimon 2006 (https://doi.org/10.1038/nature04371) and in Ohlstein and Spradling 2006 (https://doi.org/10.1038/nature04333). Anti-horseradish peroxidase (HRP) Rhodamine (TRITC)-conjugated (Jackson ImmunoResearch, 123-025-021) was show to label Drosophila gut stem cells and enteroblasts in O'Brien et al. 2011 (https://doi.org/10.1016/j.cell.2011.08.048). Goat anti-GFP (Abcam, ab5450) and rabbit anti-mCherry (Abcam, ab167453) were used as tested by the manufacturer.

Animals and other research organisms

Policy information about [studies involving animals](); [ARRIVE guidelines]() recommended for reporting animal research, and [Sex and Gender in Research]()

Laboratory animals

Adult Drosophila melanogaster, males and females, upto 2-5 weeks old. Strains used: Hand-Gal4[MI04106-TG4.0] (BDSC: 66795), mex1-Gal4, esg-Gal4 (NP7397), btl-Gal4 (DGGR: 109128), trh-Gal4 (GMR14D03, BDSC: 47463), vm-Gal4 (GMR13B09, BDSC: 48547), DSRF-Gal4 (BDSC: 25753), bnl-Gal4[MI00874-TG4.1] (this study), bnl[lexA] (a gift from Sougata Roy), QF6 (a gift from Julia Cordero), UAS-traRNAi.TRiPJF03132 (BDSC: 28512), UAS-traRNAi.GD764 (VDRC: 2560), UAS-SxlRNAi.TRiPGL00634 (BDSC: 38195), UAS-bnlRNAi.GD3070 (VDRC: 5730), UAS-btlRNAi.KK100331 (VDRC: 110277), UAS-Bax (a gift from Julia Cordero), UAS-myr(src)::GFP M7E (BDSC: 5432), UAS-StingerGFP (BDSC:84278), UAS-Flybow.1.1B (used as 10xUAS-CD8::GFP, BDSC: 56803), QUAS-mtdTomato-3xHA (BDSC: 30005), 13xlexAop2-IVS-myr::GFP (BDSC: 32209), OregonR, w1118 (GD control, VDRC: 60000), UAS-mCherryRNAi.Valium10 (TRiP control, BDSC: 35787), ovoD1 (BDSC: 1309), UAS-Dcr-2 (BDSC: 24646, 24650), UAS-Gal80TS (ref. 73, BDSC: 7108), UASp-Sxl.alt5-C8 (used as UAS-Sxl, BDSC: 58484), Ubi-EGFP.ODD, Ubi-mRFP.nls (BDSC:86536), Ldh::GFPYD0852 (a gift from U. Banerjee).

Wild animals

This study did not involve wild animals.

Reporting on sex

Drosophila virgin male and virgin female samples were used and sex is clearly stated in main text and figures. All data display and analysis is shown seperately for each sex

Field-collected samples

The study did not involve samples collected in the field.

Ethics oversight

No ethical approval was required. The use of Drosophila melanogaster does not require ethical approval or guidance.

Note that full information on the approval of the study protocol must also be provided in the manuscript.
